# Description and comparison of the skin and ear canal microbiota of non-allergic and allergic German shepherd dogs using next generation sequencing

**DOI:** 10.1371/journal.pone.0250695

**Published:** 2021-05-03

**Authors:** Neoklis Apostolopoulos, Stefanie P. Glaeser, Ruchi Bagwe, Stefan Janssen, Ursula Mayer, Christa Ewers, Peter Kämpfer, Reto Neiger, Nina Thom

**Affiliations:** 1 Department of Dermatology, Small Animal Clinic—Internal Medicine, Justus Liebig University, Giessen, Germany; 2 Institute for Applied Microbiology, Justus Liebig University Giessen, Giessen, Germany; 3 Algorithmic Bioinformatics, Justus Liebig University Giessen, Giessen, Germany; 4 Department of Dermatology, Small Animal Clinic AniCura Kleintierspezialisten Augsburg GmbH, Augsburg, Germany; 5 Institute for Hygiene and Infectious Diseases of Animals, Giessen, Germany; 6 IVC Evidensia DACH, Munich, Germany; Leibniz Institute for Zoo and Wildlife Research (IZW), GERMANY

## Abstract

Atopic dermatitis is one of the most common skin diseases in dogs. Pathogenesis is complex and incompletely understood. Skin colonizing bacteria likely play an important role in the severity of this disease. Studying the canine skin microbiota using traditional microbiological methods has many limitations which can be overcome by molecular procedures. The aim of this study was to describe the bacterial microbiota of the skin and ear canals of healthy non-allergic and allergic German shepherd dogs (GSDs) without acute flare or concurrent skin infection and to compare both. Bacterial 16S rRNA gene amplicon sequence data revealed no differences of bacterial community patterns between the different body sites (axilla, front dorsal interdigital skin, groin, and ear canals) in non-allergic dogs. The microbiota at the different body sites of non-allergic GSDs showed no significant differences. Only for the samples obtained from the axilla the bacterial microbiota of allergic dogs was characterized by a lower species richness compared to that of non-allergic dogs and the bacterial community composition of the skin and ear canals of allergic dogs showed body site specific differences compared to non-allergic dogs. Actinobacteria was the most abundant phylum identified from the non-allergic dogs and Proteobacteria from allergic dogs. *Macrococcus* spp. were more abundant on non-allergic skin while *Sphingomonas* spp. were more abundant on the allergic skin. Forward step redundancy analysis of metadata indicated that the household the dogs came from had the strongest impact on the composition of the skin microbiome followed by sex, host health status and body site.

## Introduction

Several next generation sequencing (NGS) studies in the last few years have shown that the skin of dogs, similar to humans, contains a higher diversity of bacterial taxa than previously believed [1–4]. Bacteria play an important role in both, health and disease, and changes in bacterial community composition of the skin are associated with many skin diseases in both humans and animals [2,5].

Canine atopic dermatitis (cAD) is a common skin disease in dogs characterized by a genetically predisposed inflammatory, IgE-associated, pruritic allergic disease, affecting certain body sites and ear canals [6,7]. CAD has been proposed as an animal model for human atopic dermatitis [8,9]. Environmental allergens are the most common cause of cAD [6]. But, food allergens (cutaneous adverse food reactions; CAFRs) can also cause identical clinical signs or be a flare factor of a cAD, making a clinical differentiation impossible [10,11]. The final diagnosis must be obtained through a systematic workup [12]. Studies in dogs have shown an association between the skin microbiota and allergic skin diseases. The skin of six allergic dogs without signs of pyoderma or *Malassezia* dermatitis revealed that there was a lower bacterial community diversity compared to the skin of 12 healthy dogs, but the bacterial community composition did not differ significantly [13]. Recently, a longitudinal study showed reduced diversity and different bacterial community composition in dogs with cAD and secondary pyoderma compared with healthy dogs [14]. After antimicrobial therapy and remission of skin lesions, the diversity was restored and the clustering difference of the bacterial communities was reduced [14]. Neither study examined the ear canal, which is a commonly affected body site in allergic dogs. The only study using NGS for evaluating the microbiota of asymptomatic ear canals of dogs with cAD showed no difference in the diversity but a significant difference in bacteria community composition [1]. All previously mentioned studies involved dogs from various breeds. It is well documented that cAD has breed predispositions and that the phenotype of the disease differs between breeds [15]. It is unclear if the different phenotypes of cAD affect the bacterial community composition between the breeds. To date no study has evaluated the microbiota of the skin and ear canal of only a single breed in both healthy and allergic skin disease states, thus minimizing potential bias due to allergy phenotype effect on the microbiota. The German shepherd dog (GSD) was chosen in our investigation because it is a high-risk breed for cAD [15], possibly due to an altered expression of the plakophilin 2 gene and other genes of the chromosome 27 [16].

The goal of the study was to describe and compare bacterial microbiomes of four body sites (axilla, front dorsal interdigital skin, groin, and ear canal) of healthy (non-allergic) and allergic GSDs using a 16S rRNA gene amplicon-based Illumina sequencing approach. We hypothesized that atopic dermatitis and/or CAFR influence the microbiota of the skin and ear canal of GSDs resulting in reduced bacterial diversity and significantly different bacterial community composition.

## Material and methods

### Study subjects

The clinical study was performed at the small animal clinic of the Justus Liebig University (JLU), Giessen, Germany. Samples were collected at the small animal clinic (JLU) during appointments specifically for this study. All owners were informed of the procedures and signed a consent form for sample collection. The Animal Welfare Committee of the Justus Liebig University of Giessen was informed about the study protocol and especially the sampling method was discussed. As there is neither pain, harm nor damage caused by gently rolling a cotton swab on skin, they assured us that ethical approval by the responsible authority is not required. Two groups of GSDs were studied. The control group included 12 GSDs without any history of allergic conditions or any clinical skin/ear canal lesions compatible with allergy (they will be referred to as "non-allergic" in the rest of the manuscript) at the time of examination and sampling. In order to minimize the risk of including a dog with subclinical allergy, only dogs older than four years were involved, as allergic conditions most often develop in young dogs from 6 months to 3 years [17]. In order to investigate a possible influence of the household conditions to the microbiota, two non-allergic GSDs per household were included. Neither systemic antibiotics nor any immunomodulatory or anti-inflammatory drugs were allowed six months prior to sampling. Bathing with shampoo and the use of ear cleaners was not allowed seven days prior to sampling. Twelve adult allergic GSDs were diagnosed with cAD, either due to cutaneous food reactions or environmental allergens. Standard diagnostic and therapeutic methods were used, including fulfillment of at least five of Favrot´s criteria and excluding other pruritic dermatosis (e.g. flea saliva hypersensitivity, sarcoptic mange) [12,18]. A combination of cAD and/or CAFRs with flea saliva hypersensitivity was allowed. Dogs with any secondary bacterial or fungal skin or ear infection were excluded. Systemic and topical antibacterial or antifungal agents were not allowed 30 and 14 days prior to sampling, respectively. Systemic administration of any immunomodulatory or anti-inflammatory drugs, with the exception of oclacitinib, were equally not allowed two months prior to sampling. Topical immunomodulatory or anti-inflammatory drugs had to be withdrawn 14 days prior to the study. Any shampooing and ear cleaners were not allowed seven days prior to sampling. All owners filled out a questionnaire regarding their dog’s housing, partner animals, current diseases and treatment, food supplements, frequency of bathing and type of shampoo. Owners of allergic dogs were asked about gastrointestinal signs and to score their dog’s pruritic signs using a pruritus visual analogue scale (pVAS), as previously validated [19,20].

### Assessment of lesions

All dogs were examined clinically and dermatologically including otoscopic examination using sterile powder-free gloves and an autoclavable metal otoscope. A validated site-specific lesion and scoring scale of lesions‘ severity, the Canine Atopic Dermatitis Extent and Severity Index (CADESI-4), was performed for the atopic dogs in order to evaluate any relationship of cutaneous microbiota and the severity of atopic dermatitis [21]. Skin and ear canal cytology was performed as described [22,23].

### Sample collection

Prior to sampling and in between sampling of the GSDs, the examination table was cleaned with PCR Clean™ Wipes (Minerva Biolabs, Berlin, Germany) according to the manufacturer´s manual, to avoid DNA cross-contamination. In order to minimize microbial contamination from the clinic´s floor, dogs were brought directly into the examination room and placed onto the examination table. After physical examination, the left axilla (A), left front dorsal interdigital region (Int), left side of the groin (L) and the left ear canal (O) were sampled. These body sites appear to be most commonly affected by atopy in this breed [15]. For sampling of microbiome studies we used 70% ethylene oxide sterilized forensic swabs with transport tube, polystyrene stem material and viscose swab material (Forensic Swab, Nr 80.629, Sarstedt, Nuembrecht, Germany) to ensure the absence of DNA contamination and to avoid cotton or wood mitochondria from the swab or the stem. The swabs were rubbed 40 times on the desired region, rotating one-quarter of the swab´s site (90°) for 10 times each. All samples were obtained in duplicates. Subsequently, a sample was taken for cytological purposes using a sterile cotton swab. The samples were transported immediately at 8°C to the Institute of Applied Microbiology, JLU Giessen. Samples for microbiome studies were stored at -20°C until further processing (DNA extraction).

### DNA extraction and 16S rRNA gene amplicon sequencing

Total DNA was extracted from DNA free swabs stored at -20°C using the NucleoSpin^®^ 96 Soil kit (96-well extraction system, Macherey Nagel AG, Oesingen, Switzerland) which can efficiently extract DNA from Gram-negative and Gram-positive bacteria including spores. According to manufacturer’s instruction using vacuum processing (NucleoVac 96 vacuum manifold, Macherey Nagel AG, Oesingen, Switzerland) with slight modifications, extraction was started using lysis buffer SL1 and afterward steps 1 to 5 were repeated with lysis buffer SL2, thus samples were lysed twice. Total DNA was eluted with 80 μL PCR water (1x 30 μL, 1x 50 μL) instead of SE buffer. DNA was quantified spectrophotometrically using a NanoDrop spectrophotometer (Thermo Scientific) and subsequently checked for the presence and amplifiability of 16S rRNA gene sequences for selected samples.

The 16S rRNA gene sequences of Bacteria were amplified for Illumina amplicon sequencing (LGC Genomics, Berlin, Germany) using a nested PCR approach with a first PCR with the primer system 341F (5′-CCTACGGGAGGCAGCAG-3´) and 1061R (5′-CRRCACGAGCTGACGAC-3´) (V3-V6) [24] (20 cycles) followed by a second PCR with primer system 515F (5′-GTGYCAGCMGCCGCGGTAA-3´)-Y and 926R (5′-CCGYCAATTYMTTTRAGTTT-3´)-jed (V4-V5) [25] (20 cycles), because according to previous studies, only a low amount of microbial DNA was detected on human skin analyzed by PCR [26,27]. For each sample, forward and reverse primers of the second PCR had the same 10-nt barcode sequences. The first round of PCR was carried out for 20 cycles, using the following parameters: 2 minutes 96°C pre-denaturation; 96°C for 15 seconds (s), 50°C for 30 s, 70°C for 90 s and primers without inline barcodes were used (341F/1061R). For the second round, 1 μl PCR product from the first PCR was used and the PCR conditions were the same as before. In this case, barcoded primers were added (515F-Y/926R-jed). DNA concentration of amplicons of interest was determined by gel electrophoresis. About 20 ng amplicon DNA of each sample were pooled for up to 48 samples carrying different barcodes. The amplicon pools were purified with one volume AMPure XP beads (Agencourt) to remove primer dimer and other small misspriming products, followed by an additional purification on MiniElute columns (Qiagen). About 100 ng of each purified amplicon pool DNA was used to construct Illumina libraries using the Ovation Rapid DR Multiplex System 1–96 (NuGEN). Illumina libraries were pooled, and size selected by preparative gel electrophoresis. Sequencing was done on a Illumina MiSeq using V3 Chemistry (Illumina). Raw sequence reads are available in the Sequence Read Archive (SRA) with BioSample Accession numbers SAMN14565128 to SAMN14565223 in the BioProject PRJNA624030.

### Amplicon sequence data analysis

The NGS analysis pipeline (https://www.arb-silva.de/ngs) of the SILVA rRNA gene database (SILVAngs 1.3) was used for sequence analysis [28]. For this, datasets of all combined sequence reads (adaptor and primer clipped) were uploaded to the database. All reads were aligned using the SILVA Incremental Aligner (SINA v1.2.10 for ARB SVN revision21008) [29], against the SILVA SSU rRNA SEED and quality controlled [28]. Reads shorter than 50 aligned nucleotides and reads with more than 2% of ambiguities, or 2% of homopolymers, were excluded from further processing. Reads with a low alignment quality (50 alignment identity, 40 alignment score reported by SINA) as well as putative contaminations and artifacts, were identified and excluded from downstream analysis. The next process step was dereplication and clustering with cd-hit-est (version 3.1.2; http://www.bioinformatics.org/cd-hit) using *accurate mode*, ignoring overhangs and applying identity criteria of 1.00 and 0.98, respectively [30]. For classification a local nucleotide BLAST search was performed against the non-redundant version of the SILVA SSU Ref dataset (release 128; http://www.arb-silva.de) using blastn (version 2.2.30+; http://blast.ncbi.nlm.nih.gov/Blast.cgi) with standard settings [31]. Unique reads were clustered in operational taxonomic unit (OTU) on a per sample basis under the criterium of 98% sequence identity to each other (pairwise distance and single linkage clustering). The longest read in each cluster was classified as the reference for each OTU and was mapped onto all reads that were assigned to the respective OTU. OTUs were assigned to taxonomic paths (genus level). Several OTUs were thereby assigned to the same taxonomic path/genus. This process resulted in quantitative information (number of individual reads of all OTUs per taxonomic path), despite the PCR limitations, possible sequencing technique biases and multiple rRNA operons. Reads without any BLAST hits or reads with weak BLAST hits, where the function “(% sequence identity + % alignment coverage)/2” did not exceed the value of 93, remained unclassified and were assigned in the virtual taxonomical group “No Relative” in the SILVAngs fingerprint and Krona charts [32], as previously reported [33,34].

Alpha and beta diversity analyses were performed in PAST3 (https://folk.uio.no/ohammer/past) [35] at the level of genera (phylogenetic groups). Alpha diversity was measured by calculating different diversity indices considering the number of phylogenetic groups per samples and the number of reads per phylogenetic group. The Shannon (overall alpha diversity) and Chao1 (richness; number of taxa corrected by the presence of singleton) indices were calculated. Significant differences between the alpha diversity indices determined for non-allergic vs allergic dogs were further evaluated. For two groups, single body sites of non-allergic versus allergic dogs, unpaired t-tests were performed in SigmaPlot 13 (Systat Software Inc.). First, a normality test (Shapiro-Wilk) was performed [36]. If the data passed the normality test, a two-tailed p-value was obtained from the t-test analysis. If the normality test failed, the Mann-Whitney Rank Sum Test was applied to test for the presence of significant differences. For more than two groups (all body sites), the significant differences of the alpha diversity values were evaluated by performing a Kruskal Wallis Test [37]. The beta diversity (comparison of the phylogenetic composition of the bacterial communities of the different samples) was studied based on relative abundance data analyzed by non-metric multidimensional scaling (NMDS; [38]) based on a Bray-Curtis similarity matrix [39]. Environmental variables, e.g. body sites, home (living in the same household) and sex, were included to the NMDS plot displayed as biplot vector. One way ANOSIMs (9999 permutations) [39] and One way PERMANOVA (9999 permutations) [40] were performed to determine significant differences (evaluating the sequential Bonferroni p-values) among samples. SIMPER (Similarity Percentage) analysis [39] also based on a Bray Curtis similarity matrix, was used to determine the average percent contribution of the different taxa to the dissimilarity among samples. Based on relative abundance patterns and contributions to community differences (SIMPER analysis) a selection of bacterial taxa was further studied with respect to significant differences in relative abundance in non-allergic vs allergic dogs. T-test were performed as described above. For correction for multiple hypothesis testing, p values were divided through the number of performed tests.

We quantified the effect size of multiple metadata fields (collection timestamp, sex, oclacitinib, host health status, host subject id) by combining them in a linear model and performed forward step redundancy analysis (RDA) as previously described [41], with the rda and ordiR2step functions in the vegan package in R 3.6.1 [42].

By systematically testing metadata fields for correlation with alpha- (using the two metrics ’Shannon’, ’chao1’) or beta-diversity (using metric ’Bray-Curtis’ [43]) via Kruskal-Wallis [44], Spearman- [45], and Pearson-correlation [46] (for alpha diversity) and PERMANOVA [40] and ANOSIM [39] (for beta diversity), we found that the following fields were significant in at least one of the tests: collection timestamp, sex, oclacitinib, host health status, host subject id and household id.

To illustrate covariance among these metadata fields, we examined the correlation between individual metadata variables. Categorical metadata fields (all but collection timestamp) were compared using a modified CramérV statistic [47]). Continuous (collection timestamp) and categorical covariates were compared with a Welchttest [47]. Resulting correlation ratios were visualized as a heat map.

## Results

### Study subjects

Overall 29 GSDs were sampled but five were excluded according to the exclusion criteria (Table 1 in S1 File). Twelve non-allergic (6 male, 1 male castrated, 4 intact female, and 1 spayed female) and twelve allergic (2 intact male, 1 male castrated, and 9 intact female) GSDs were further analyzed (Table 1 in S1 File). The dogs were living in a radius of max 131 kilometers away from Giessen (Germany). The age of the non-allergic dogs (mean: 7.5 ± 1.9 years) was significantly higher (t-Test, p< 0.01) than the age of allergic GSDs (mean: 4.8 ± 2.2 years) (Table 2 in S1 File). All allergic dogs had mild lesions with a median CADESI-04 score of six (range 0–11) and a median PVAS score of four (range 0–8). Two dogs had a PVAS score of 7/10 and 8/10, respectively, with only mild skin lesions. Six allergic GSDs received labeled doses of oclacitinib (Apoquel, Zoetis Deutschland GmbH, Berlin, Germany).

### Analysis of the phylogenetic composition of the GSDs microbiota

In total, 4,038,850 paired end sequences with an average sequence length of 373 nucleotides (nt) were obtained. Briefly, 2,579 sequences (0.06%) were rejected because they failed the SILVAngs pipeline quality control. Finally, 4,036,271 sequences (2,334–246,525 per sample) were further analyzed (Table 3 in S1 File). In total 241,114 unique reads (5.97% of the finally analyzed sequences) were assigned to OTUs. Additionally, 1,129,221 (27.96%) sequences (number of sequence reads with 98% sequence identity to each other; defined as "clustered"), and 2,665,936 (66.01%) sequences (number of sequence reads with 100% identity to another; defined as "replicates") were assigned to the OTUs. Each OTU was classified in the SILVA database with a taxonomic paths with maximum resolution at the genus level (named as ´phylogenetic groups`for unnamed genera). Chloroplast, mitochondrial, and archaeal 16S rRNA gene sequences were detected with a relative abundance of 20.13% (190,605 sequences), 0.07% (9,430 sequences), 0.02% (1,064 sequences) of the total analyzed sequences, respectively (Table 3 in S1 File). Sequences that did not match any known taxa (sequence similarity <93% to the next known taxon) were classified as “no relative” and had 0.3% relative abundance (5,131 sequences) (Table 3 in S1 File). Sequences assigned to *Archaea*, chloroplasts, mitochondria, and no relative groups were excluded from further analyses. Only sequences assigned to the domain Bacteria (3,830,041 in total; 2,334 to 232,445 per sample) were further analyzed and were set to 100% (per sample).

### Skin Microbiome of non-allergic German shepherd dogs

#### Diversity and richness of bacterial communities at skin samples of non-allergic GSDs

**Fig 1:**
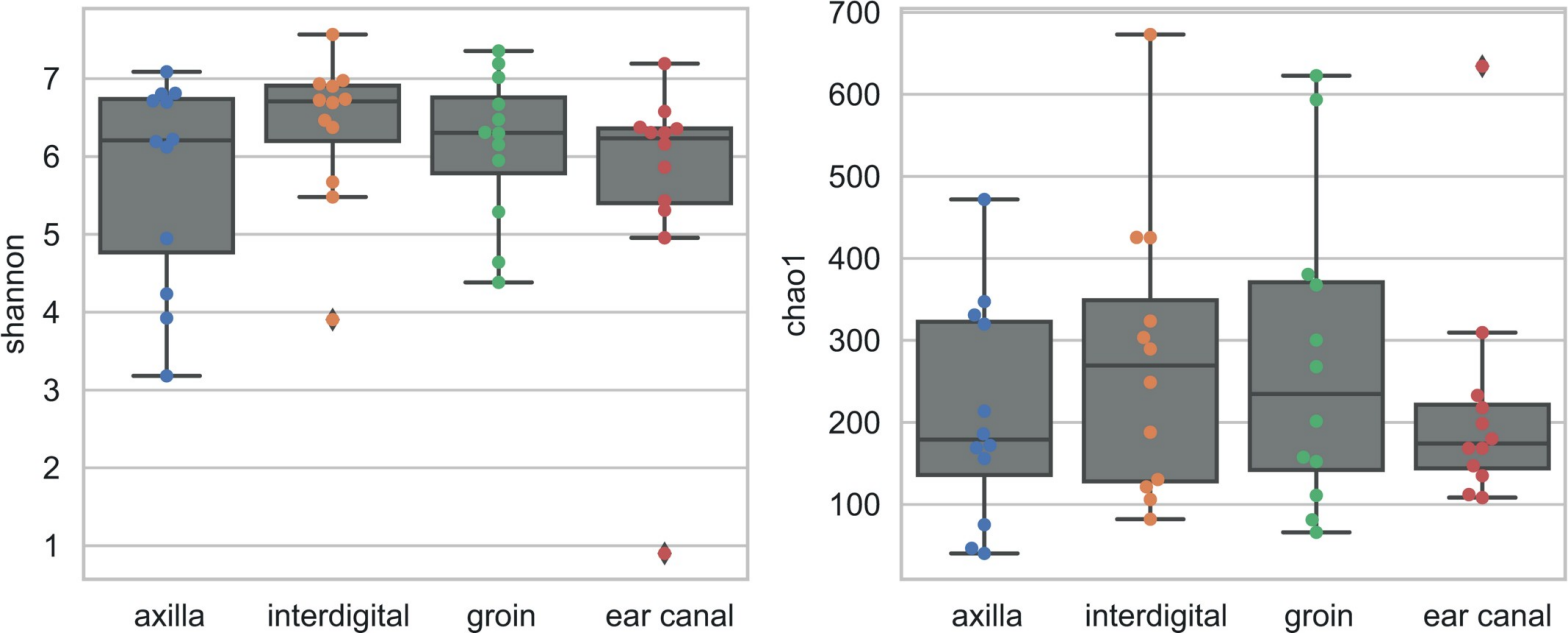
Box-plots of alpha diversity indices Shannon (a) and Chao1 (b) for the four studied body sites of non-allergic dogs. No significant differences were obtained between the body sites using Kruskal-Wallis tests.

Diversity (Shannon index) and richness (Chao1) of phylogenetic groups (genera) at the four body skin sites (axilla, interdigital, groin, ear canal) were not significantly different between the body sites (Kruskal-Wallis for Shannon index, p = 0.6214; for Chao1, p = 0.4784) (Fig 1 and Fig 1 in S2 File).

However, a greater number of individual samples from the interdigital region and ear canal had a more homogeneous composition of the microbiota, showing a lower richness (Chao1 values; Fig 1 in S2 File).

#### Skin microbial composition of non-allergic GSDs

**Fig 2:**
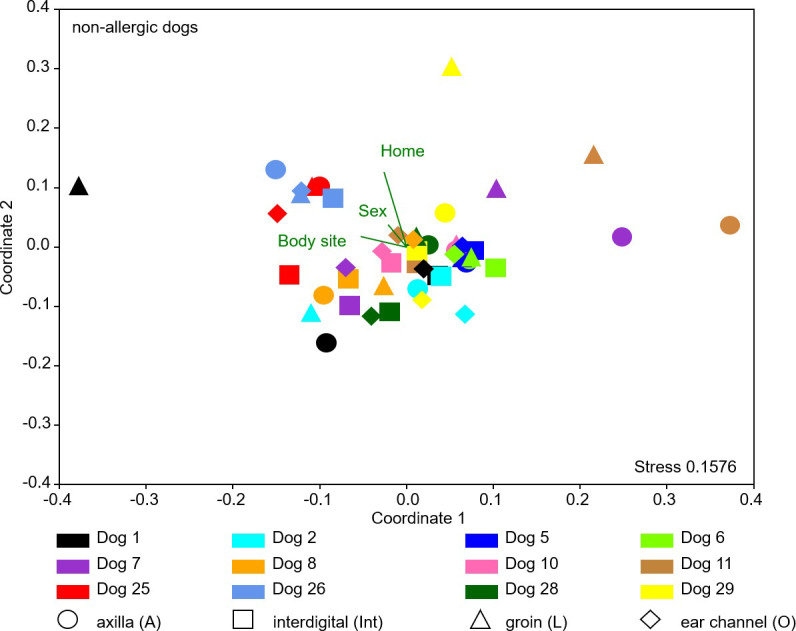
Comparative analysis of the bacterial community composition of the skin microbiota of all non-allergic dogs. Comparative analysis of the relative abundance patterns of the skin microbiota composition of all body sites (different objects), performed by NMDS analysis based on a Bray-Curtis similarity matrix. Sex, body site, and home were included as environmental parameters (biplots). Different colors represent individual dogs.

Bacterial community patterns present at the different body sites for the individual non-allergic dogs were inspected by NMDS analysis based on a distance matrix generated with the Bray Curtis beta diversity metric. One way ANOSIM analysis (p = 0.17) and One way PERMANOVA analysis (p = 0.1808) did not reveal significant differences between the four different body sites (axilla, interdigital, groin, and ear canal) of the non-allergic GSDs. A variability across all dogs and all different body sites was documented. The factor home had the strongest impact on the bacteria community composition (largest relative length of biplot vector; Fig 2). Significant differences in community composition were identified when grouping by sex (male versus female; One way ANOSIM and One way PERMANOVA p< 0.001; R = 0.1337) was conducted. Significant differences in the bacterial community composition were also observed when grouped by homes (both One way ANOSIM and One way PERMANOVA p< 0.003; R = 0.1337; Fig 2 in S2 File; homes Table 1 in S1 File).

#### Composition (most abundant taxa) of the skin of non-allergic GSDs

**Fig 3:**
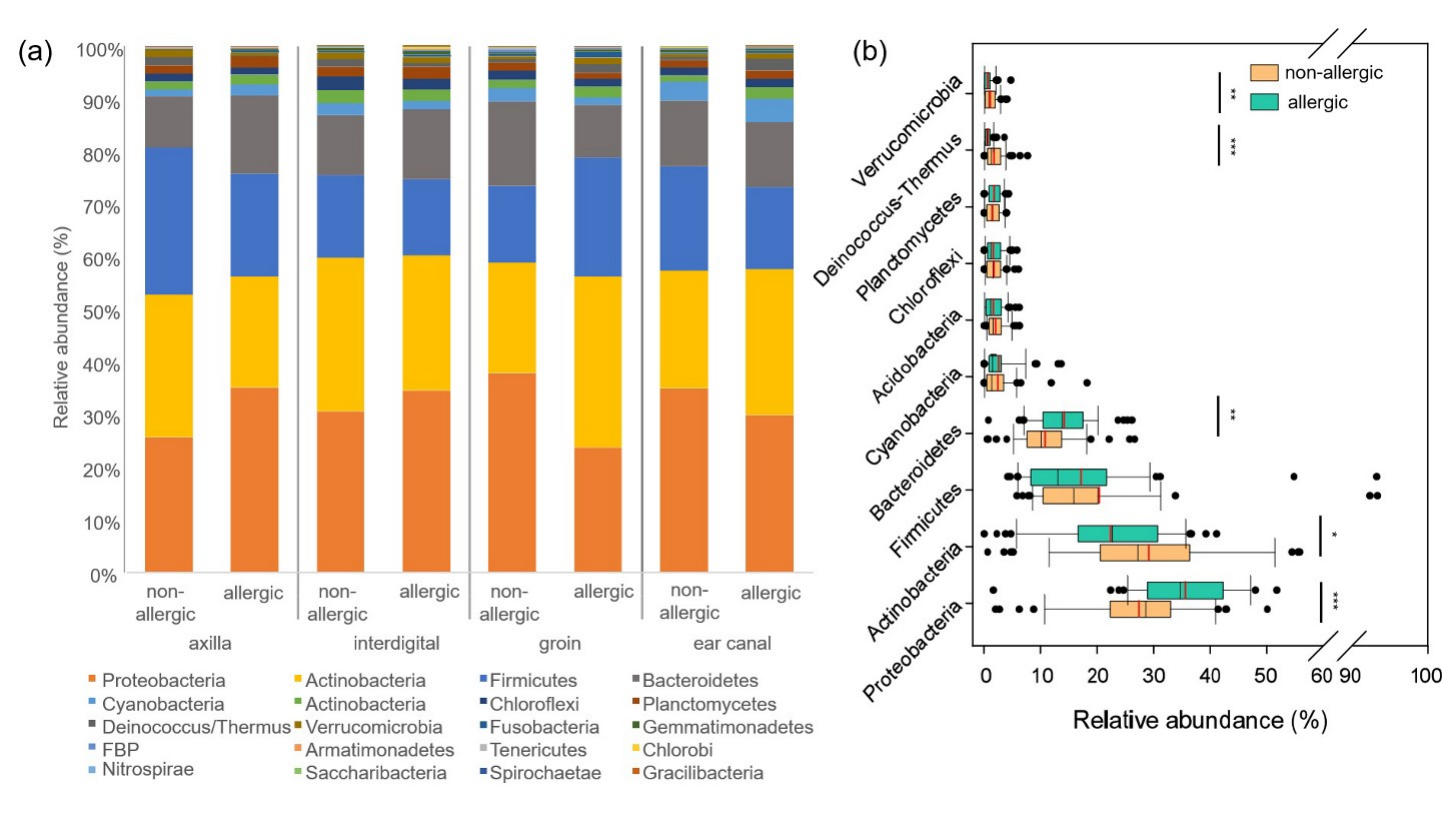
Phylogenetic composition of the skin (axilla, interdigital, groin) and ear canal microbiota of non-allergic (n = 12) versus allergic (n = 12) GSDs resolved at the phylum level. (a) Mean relative abundances of bacterial phyla identified in axilla, interdigital, groin and ear canal of non-allergic and allergic dogs. (b) Variation of the relative abundances (across all body sites) of the 10 most abundant phyla among non-allergic and allergic dogs. Asterisks are representing statistical significance: * p< 0.05; ** p< 0.01; *** p< 0.001.

Forty-four phyla in total were identified from the four sample sites (axilla, interdigital, groin, ear canal) taken from the non-allergic dogs, of which 30 phyla had a relative abundance lower than 1% (Table 4 in S1 File). The ten most abundant phyla of non-allergic dogs were Actinobacteria (mean relative abundance 29.1 ± 14.7%) followed by Proteobacteria (27.4 ± 10.1%), Firmicutes (20.3 ± 18.7%), and Bacteroidetes (10.8 ± 5.4%), Cyanobacteria, Acidobacteria (both 2.0 ± 1.6%), Chloroflexi (1.8 ± 1.6%), Planctomycetes (1.7 ± 1.4%), Deinococcus-Thermus (1.5 ± 1.2%), and Verrucomicrobia (1.1 ± 1.0%) (Fig 3A and 3B). In samples from the interdigital region and the ear canal Proteobacteria were most abundant followed by Actinobacteria, Firmicutes, Bacteroidetes, and Cyanobacteria. Groin samples were dominated by Actinobacteria followed Proteobacteria, Firmicutes, Bacteroidetes, and Cyanobacteria. Ιn the samples from the axilla Firmicutes was most abundant followed by Actinobacteria, Proteobacteria, Bacteroidetes, and Cyanobacteria (Fig 3A and Table 4 in S1 File).

**Fig 4:**
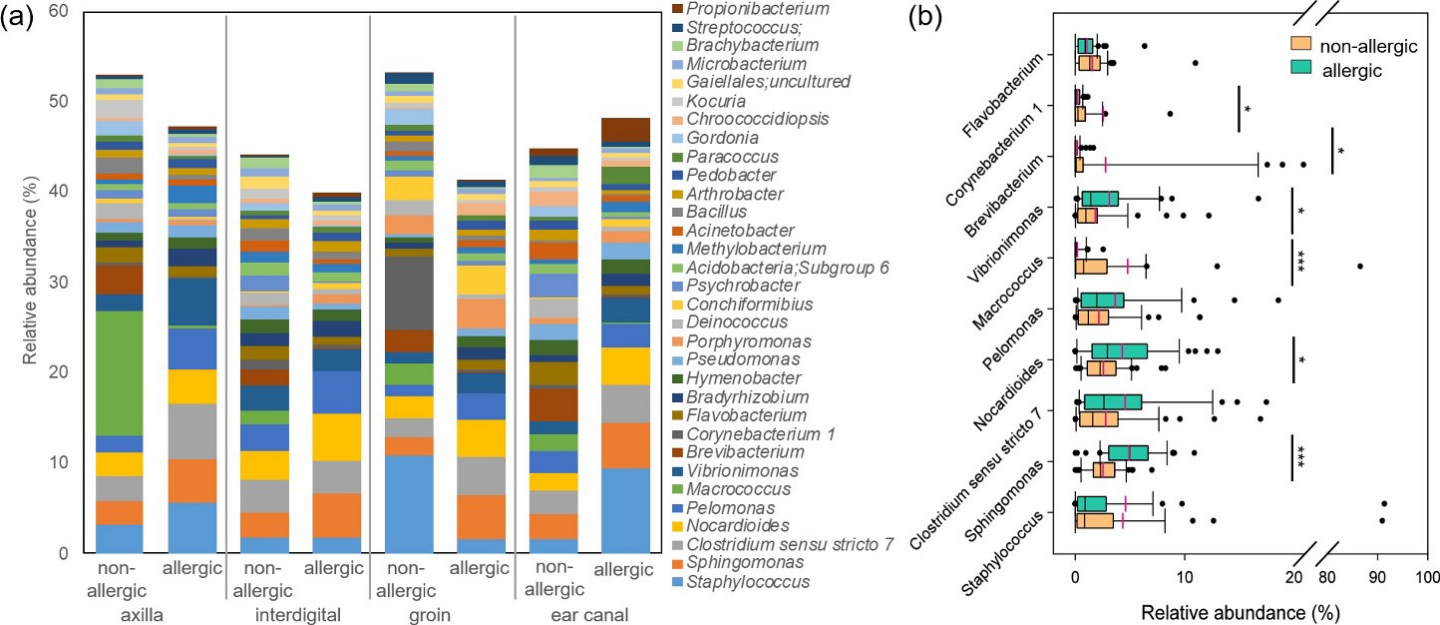
Phylogenetic composition of the skin (axilla, interdigital, groin) and ear canal microbiota of non-allergic (n = 12) versus allergic (n = 12) GSDs resolved at the genus level. (a) Mean relative abundances of bacterial genera identified in axilla, interdigital, groin and ear canal of non-allergic and allergic dogs. All genera with a total relative abundance of >5% are depicted. (b) Relative abundance (across all body sites) of the 10 most abundant genera of non-allergic and allergic dogs. Asterisks are representing statistical significance: * p< 0.05; ** p< 0.01; *** p< 0.001.

Relative abundance patterns of genera are given in Table 5a and 5b in S1 File. Analysis of the aggregated mean relative abundances showed that the genera *Macrococcus* and *Staphylococcus* were most abundant (> 4%) at the skin of non-allergic dogs (Fig 4 and Tables 5b, 6a and 6b in S1 File). Body site specific differences were obtained (Fig 4A and Tables 5b and 7 in S1 File). The skin of the axilla was mostly colonized by *Macrococcus* (mean relative abundance 13.8 ± 29.8%) followed by *Brevibacterium*, *Staphylococcus*, *Clostridium sensu stricto* 7, *Nocardioides*, *Sphingomonas*, and others. The interdigital area was dominated by *Clostridium sensu stricto 7* (mean relative abundance 3.5 ± 2.7%) followed by *Nocardioides*, *Pelomonas*, *Sphingomonas*, *Vibrionimonas*, *Psychrobacter*, *Bravibacterium*, and *Staphylococcus*. The groin was mostly colonized by *Staphylococcus* (mean relative abundance 10.8 ± 25.5%), followed by *Corynebacterium* 1, *Conchiformibius*, *Nocardioides*, *Brevibacterium*, *Macrococcus*, and *Porphyromonas*. *Brevibacterium* had the highest mean relative abundance (3.7 ± 7.6%) in the ear canal followed by *Clostridium sensu stricto* 7, *Sphingomonas*, *Psychrobacter*, *Pelomonas*, *Flavobacterium*, *Deinococcus*, and *Pseudomonas*. Subsequently, each of the four taxa with the highest mean relative abundance per body site (axilla: *Macrococcus;* interdigital: *Clostridium sensu stricto* 7; groin: *Staphylococcus;* ear canal: *Brevibacterium*) was evaluated individually for significant different abundance between the four body sites. None of these taxa had a significantly different relative abundance between the four body sites (normality failed; Kruskal-Wallis-test for axilla vs interdigital vs groin vs ear canal; *Macrococcus*: p = 0.975; *Clostridium sensu stricto 7*: p = 0.085; *Staphylococcus*: p = 0.288; *Brevibacterium*: p = 0.406).

### Skin microbiome of allergic German shepherd dogs

#### Diversity and richness of bacterial communities at skin samples of allergic GSDs

The diversity (Shannon index) and richness (Chao1) of phylogenetic groups at the four body sites (axilla, interdigital, groin, ear canal) of allergic GSDs showed no significantly different between the body sites (Kruskal-Wallis for Shannon index, p = 0.334; for Chao1, p = 0.78) (Fig 3 in S2 File).

#### Skin microbial composition of allergic GSDs

The bacterial community patterns of the different body sites for the individual allergic dogs were investigated by NMDS analysis based on a similarity matrix generated with the Bray Curtis similarity index. One way ANOSIM analysis (p = 0.6) and One way PERMANOVA analysis (p = 0.32) did not reveal significant differences between the four different body sites (axilla, interdigital, groin, and ear canal) of the allergic GSDs (Fig 4 in S2 File).

**Fig 5:**
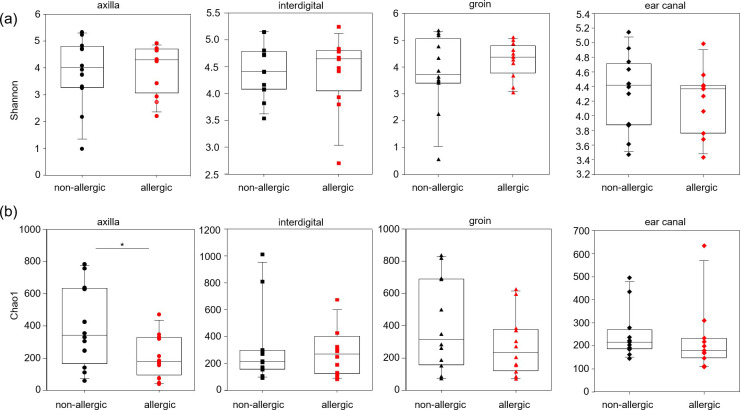
Box-plots of the alpha diversity indexes Shannon (total diversity) (a) and Chao1 (b) of the skin microbiota per body site (axilla, interdigital, groin, ear canal) of non-allergic and allergic German shepherd dogs, based on Illumina 16S rRNA gene amplicon sequencing of microbial communities. Asterisks are representing statistical significance: * p < 0.05.

In order to evaluate if oclacitinib had an effect in the composition of the skin microbiota of the allergic dogs, we compared the bacterial community compositions, as described above for the subgroups allergic with oclacitinib (n = 6) and without oclacitinib (n = 6) (Fig 5 in S2 File). No significant difference was obtained, proposing that oclacitinib did not affected the cutaneous and ear canal microbiota composition (One way ANOSIM, p-values: axilla: p = 0.2777; interdigital: p = 0.2949; groin: p = 0.1494; ear canal: p = 0.9022) among the allergic dogs, given the small number of the subgroup population. On this basis we assume that oclacitinib should not affect the comparison between allergic and non-allergic dogs.

#### Composition (most abundant taxa) of the skin of allergic GSDs

Proteobacteria (mean relative abundance 35.5 ± 10.9%) and Actinobacteria (22.4 ±10.2%) were the most abundant phyla of the allergic GSDs and dominated in each of the four sample sites (Fig 3A and 3B and Table 8 in S1 File). They were followed by Firmicutes (17.0 ± 14.6%), Bacteroidetes (14.1 ± 5.2%), Cyanobacteria (2.5 ± 3.0%), Planctomycetes (1.8 ±1.2%), Chloroflexi (1.7 ± 1.5%), Acidobacteria (1.7 ± 1.6%), Verrucomicrobia (0.7 ± 0.9%), and Deinococcus-Thermus (0.6 ± 0.7%).

Overview of relative abundance patterns of genera present in the microbiota at different body sites of allergic dogs is given in Table 5a and 5b in S1 File. Aggregated mean relative abundance data showed that *Sphingomonas*, *Staphylococcus*, *Clostridium sensu stricto* 7, and *Nocardioides* dominated the skin microbiota of allergic GSDs (each genus > 4% aggregated relative abundance) (Fig 4 and Tables 5b, 6a and 6b in S1 File). Analysis of the mean relative abundances at the genus level revealed some body site specific differences (Fig 4A and Tables 5b and 7 in S1 File). *Clostridium sensu stricto* 7 (mean relative abundance 6.2 ± 7.8%) was the most abundant phylogenetic group on the skin of the axilla, followed by *Staphylococcus*, *Vibrionimonas*, *Sphingomonas*, *Pelomonas*, and *Nocardioides*. The interdigital skin was dominated by *Nocardioides* (mean relative abundance 5.3 ± 4.4%), followed by *Sphingomonas*, *Pelomonas*, *Clostridium sensu stricto 7*, *Vibrionimonas*, *Bradyrhizobium*, *Staphylococcus*, and *Hymenobacter*. The skin of the groin was dominated by *Sphingomonas* (mean relative abundance 4.9 ± 2.4%), followed by *Clostridium sensu stricto 7*, *Nocardioides*, *Porphyromonas*, *Conchiformibius*, *Pelomonas*, and *Vibrionimonas*. Finally, the ear canal of the allergic GSDs was dominated by *Staphylococcus* (mean relative abundance 9.4 ± 25.9%), followed by *Sphingomonas*, *Clostridium sensu stricto* 7, *Nocardioides*, *Vibrionimonas*, *Pelomonas*, *Propionibacterium*, and *Paracoccus*.

### Comparative analysis of the skin microbiome of non-allergic versus allergic German shepherd dogs

#### Differences in diversity and richness of skin microbiota of non-allergic versus allergic GSDs

Comparing the diversity between non-allergic and allergic GSDs no significant differences were obtained for the Shannon index for each body site (Table 9 in S1 File and Fig 5). A significantly lower bacterial community richness (Chao1 index) was only determined at the skin of the axilla of allergic GSDs (A; Chao1 index; p = 0.032; Table 9 in S1 File and Fig 5).

#### Differences in the composition of bacterial communities of non-allergic versus allergic GSDs

**Fig 6:**
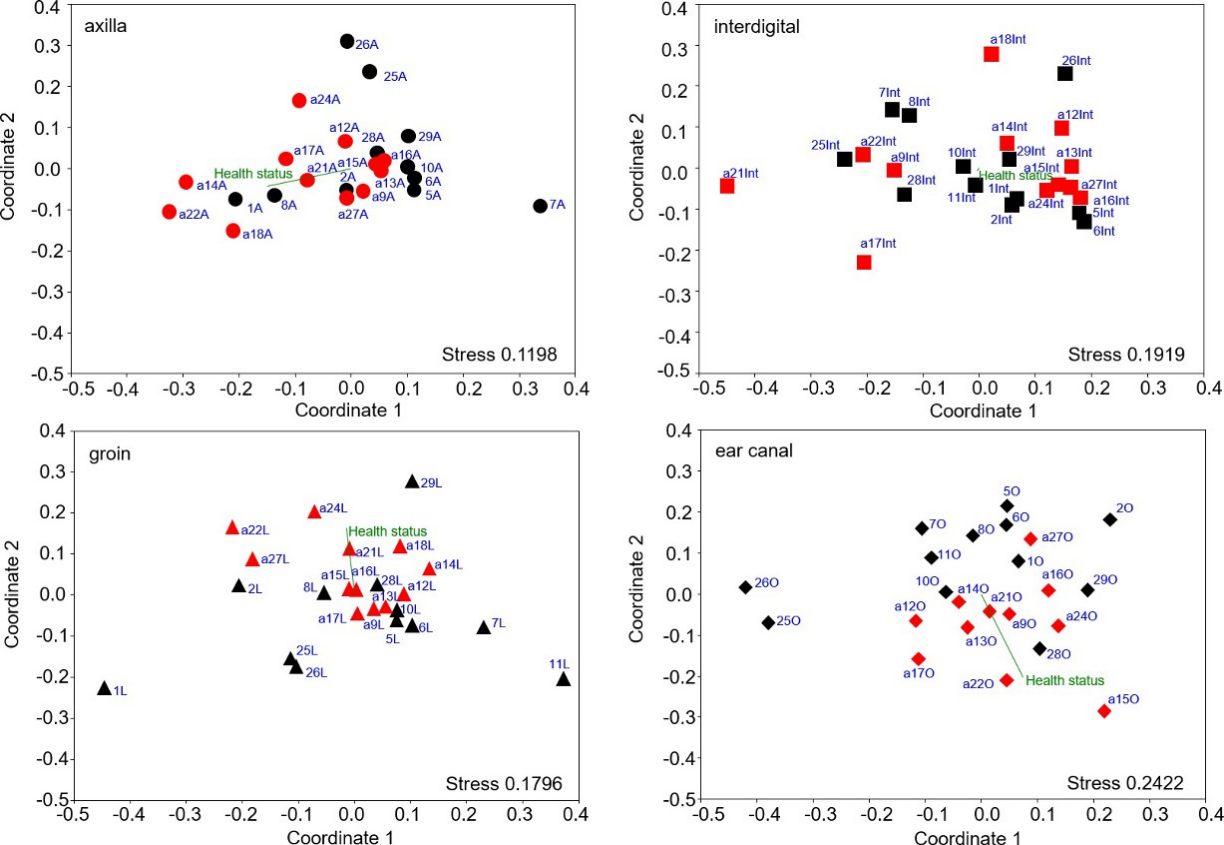
Comparative analysis of the skin microbiota composition of relative abundance patterns at each body site of non-allergic (n = 12; black color) and allergic (N = 12; red color) dogs, performed by NMDS analysis based on a Bray-Curtis similarity matrix. The health status was included as environmental parameter; (A- Axilla, Int- interdigital area, L- groin, O- ear canal).

Shifts in the bacterial community composition at the skin of allergic dogs compared to non-allergic dogs were visualized in NMDS plots (Fig 6). Significant differences were obtained for the axilla (A; One way ANOSIM p = 0.048, R = 0.1001; One way PERMANOVA p = 0.0256), groin (L; One way ANOSIM p = 0.036, R = 0.00386; One way PERMANOVA p = 0.0264) and ear canal (O; One way ANOSIM p = 0.0012, R = 0.1842; One way PERMANOVA p = 0.0025), but not for the interdigital area (Int; One way ANOSIM p = 0.1548; One way PERMANOVA p = 0.1171). The inclusion of the health status (non-allergic vs allergic) confirmed that allergy correlated with the significant differences of the clustering of the community patterns between the samples (Fig 6). Sample a18O (ear canal of the allergic dog a18) was excluded from the NMDS plot because the community was too different to visualize minor differences among the other samples more clearly (Fig 6 in S2 File). The strong difference of the community profile was due to the high relative abundance of the genus *Staphylococcus* (91.3%) in this skin sample compared to 0.02–6.4% in the other skin samples (Table 5 in S1 File).

In order to determine which taxa (phylogenetic groups/genera) had the main contribution to the community differences among non-allergic versus allergic GSD in general and for each body site specifically, SIMPER analyses were performed (Tables 6a and 10 in S1 File). *Staphylococcus* and *Macrococcus* had with 4.89% and 4.8% the highest contribution to general differences between the colonization of non-allergic versus allergic dogs if aggregated rel. abundance patterns of bacterial genera were compared (SIMPER analysis; Table 6 in S1 File). Genera with the highest contributions to community differences among non-allergic and allergic dogs (>2%) for individual body sites are listed in Table 10 in S1 File. The genus *Macrococcus* (9.0% contribution) was the major contributor for the differences in the samples from the axilla (A), followed by *Staphylococcus* (4.7% contribution). For the samples from the groin (L) the genus *Staphylococcus* contributed the most (7.0% contribution). For the ear canal (O), *Brevibacterium* contributed the most to the differences (2.5% contribution) with *Staphylococcus* of the sample a18O being excluded as dominated all the other, as mentioned above, affecting the comparison.

#### Composition (most abundant taxa) of the skin of non-allergic versus allergic GSDs

The order of the 10 most abundant phyla of allergic GSDs was different compared with non-allergic GSDs (Tables 4 and 8 in S1 File; Fig 3). Between the two groups, Proteobacteria (p< 0.001) and Bacteroidetes (p = 0.003) had a significantly higher mean relative abundance at the skin of allergic dogs, whereas Actinobacteria (p = 0.012), Deinococcus-Thermus (p< 0.001) and Verrucomicrobia *(*p = 0.016) occurred in a significantly lower relative abundance in allergic dogs. Firmicutes showed no significant differences between allergic and non-allergic GSDs (Fig 3B).

Evaluating the mean relative abundances of the phylogenetic groups for significant differences between non-allergic and allergic GSDs showed that *Sphingomonas* was significantly higher abundant in allergic GSDs (p<0.001; mean rel. abundance of non-allergic 2.5 ± 1.4% versus 4.9 ± 2.3% of allergic dogs), as well as *Nocardioides* (p = 0.034; mean rel. abundance of 2.5 ± 1.8% for non-allergic dogs versus 4.3 ± 3.4% for allergic dogs) (Fig 4B). No significant difference was observed for *Staphylococcus* (p = 0.8, Table 11a in S1 File) and *Clostridium sensu stricto* 7 (p = 0.062) between non-allergic and allergic GSDs. Interestingly, allergic dogs had significantly lower mean relative abundance of *Macrococcus* (p< 0.001; mean rel. abundance of 4.8 ± 15.5% for non- allergic dogs versus 0.1 ± 0.4% for allergic dogs; Table 11b in S1 File) and *Brevibacterium* (p = 0.016; mean rel. abundance of 2.7 ± 6.3% for non- allergic dogs versus 0.1 ±0.3% for allergic dogs) than non-allergic GSDs.

A more detailed comparison between non-allergic and allergic GSDs of the most abundant taxa per body site revealed important findings (Fig 4). *Clostridium sensu stricto 7* occurred in a significantly higher relative abundance on the axilla of allergic dogs (p = 0.026; mean relative abundance of 2.7 ± 5.0% for non-allergic dogs versus 6.2 ± 7.8% for allergic dogs). *Nocardioides* of the interdigital skin showed no significant difference (p = 0.138). *Sphingomonas* was significantly more abundant in the groin of the allergic dogs (p = 0.002; mean relative abundance of 2.0 ± 1.5% for non-allergic dogs versus 4.9 ± 2.4% for allergic dogs). In addition, *Sphingomonas* was significantly more abundant in the non-allergic versus allergic GSDs for multiple sites (axilla: p = 0.013; interdigital: p = 0.014; ear canal: p = 0.017, Table 7 in S1 File). *Staphylococcus* did not show any significant difference for any site between the allergic and non-allergic GSDs (Table 11a in S1 File). On the contrary, a significantly lower relative abundance of *Macrococcus* was obtained from samples of the interdigital skin, the groin, and the ear canal of the allergic dogs but there was no significant difference for the axilla (Table 11b in S1 File). In addition, *Brevibacterium* was also evaluated for significant difference for the ear canal samples because it was the main contributor to the difference of the bacterial community composition between the two groups in SIMPER analysis (Table 10 in S1 File), with the allergic group having a significantly lower mean relative abundance (p = 0.041; mean relative abundance in ear channel microbiota (Table 7 in S1 File) of 3.6 ± 7.7% for the non-allergic dogs versus 0.1 ± 0.4% for the allergic dogs).

### Evaluation of the impact of metadata on the alpha and beta diversity analysis of skin microbiota

**Fig 7:**
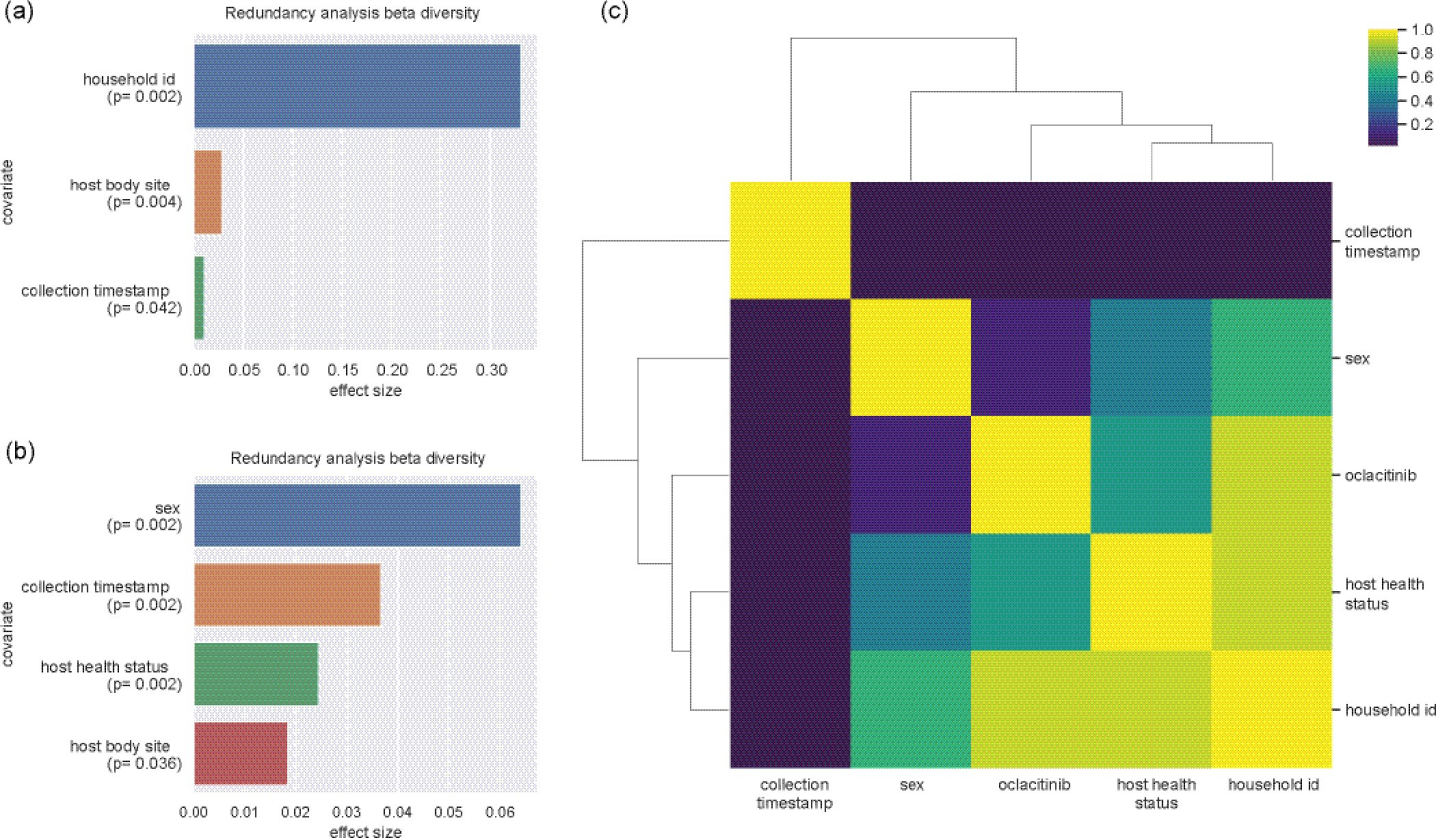
Forward step redundancy analysis (RDA) indicating the impact of metadata on beta diversity analysis including all studied dogs and body skin sites (a; b, excluding the factor household) and heatmap showing the interrelation of metadata (c).

Finally, the impact of metadata (collection timestamp, sex, oclacitinib, host health status, host subject id) on alpha and beta diversity data across all studied dogs and body sites was evaluated by RDA analysis. We found that the following fields were significant in at least one of the tests: collection timestamp, sex, oclacitinib, host health status, host subject id, and household id. Alpha diversity index values (Shannon, Chao1) were only significantly impacted by the factor household (Fig 7 in S2 File) and if this factor was excluded only sex of the dogs showed a significant contribution on alpha diversity (Fig 7 in S2 File). A higher impact of different factors was obtained for the beta diversity. Here again the household had the predominant impact on community differences in the skin microbiota (Fig 7A). Lower significant impact was observed for the factors host body site and the collection time stamp. If the factor household was excluded from RDA analysis for beta diversity, sex, followed by collection timestamp, dog health status and host body site had in decreasing order an impact on differences in bacterial community composition (Fig 7B). The interrelationship of the different metadata is illustrated in Fig 7C. Household combined with host subject ID (individual dogs), host health status and oclacitinib treatment had the strongest interrelationships and effects on skin microbiota community shifts.

## Discussion

In this NGS study, we described the bacterial community of the skin and ear canal of non-allergic GSDs and compared it to the skin and ear canal microbiota of allergic GSDs. Several NGS studies have also examined the canine skin or ear canal in health and disease. These studies used different techniques, from sample collection and storage, to DNA extraction and analysis and as shown before, methodology influences results [48–50]. Therefore, a direct side by side comparison of studies is difficult [48]. Nevertheless, any of those results are useful to further characterize the canine cutaneous microbiota in health and disease. Our 16S rRNA gene amplicon approach (nested PCR; V3-V6 & V4-V5) showed high individual and body site variability between the different study subjects, without significant differences between the body sites, in contrast to the first NGS-based study in veterinary medicine [13]. In our study the body skin (axilla, interdigital, groin) showed significantly higher species richness than the ear canal in non-allergic dogs. Possible explanations may include the special ear canal construction (chamber-like) and histology, with a comparatively thin epidermis and dermis containing single hair follicles, sebaceous glands and ceruminal glands, providing lipid-rich cerumen determining it´s microenvironment with a relatively high humidity [51,52]. Bactericidal activity of cerumen could explain the lower species richness compared to the other body sites.

We identified Actinobacteria as the most dominant phylum on the non-allergic dogs, similar to another study [53]. This finding is also in line with the bacterial composition of human skin [3]. In contrast, the first NGS veterinary study describing healthy canine cutaneous bacterial composition showed predominately Proteobacteria [13]. As mentioned above, different methodology could explain such differences as studies have shown [48,50,51,54]. Thus, this phenomenon is not unique to dogs. Furthermore, studies have shown that environmental factors can influence the skin microbiome in humans like contact with soil or plant material [53,55–57]. Therefore, it can be assumed that different habits or environment of the dogs could explain differences in results. In addition, temporal changes of microbiome can also influence the results and lead to such disagreements between studies. To date temporal stability of cutaneous microbiome in animals remains largely unknown. In rats healthy skin showed temporal instability (11 days) [58], whereas the healthy canine ear canal remained stable for 28 days. [1]. Both, living in the same household and sex affected the bacterial community patterns among the samples of the healthy dogs, in agreement with other studies [53,59]. Interestingly in our study, Proteobacteria predominated in the ear canal and the interdigital area, as in another study for the interdigital area and the pinna [13]. It is not clear why these two areas showed this difference in comparison with the groin and the axilla of the GSDs. One possible explanation for the interdigital area could be direct and repeated contact with soil, where it is shown that Proteobacteria predominate its bacterial composition [60]. Currently there are only two other NGS studies describing the canine ear canal. In the first study, Proteobacteria were the most abundant phyla followed by Actinobacteria, Firmicutes, Bacteroidetes, and Fusobacteria with *Escherichia* as the most abundant genus [1]. The second study showed also similar most abundant phyla but in a different order, with Firmicutes being most abundant followed by Proteobacteria, Bacteroidetes, and Actinobacteria with *Romboutsia* as the most abundant genus [61]. Our findings were consistent with the first study [1], as we had the same order of the most common phyla, except that we documented Cyanobacteria instead of Fusobacteria. At the genus level we identified *Brevibacterium* as the most abundant taxon. The most abundant genus found on non-allergic skin was *Macrococcus* with the highest abundance in the axilla. The second most common genus was *Staphylococcus* with the highest abundance in the groin followed by the axilla, the interdigital area, and the ear canal. *Clostridium sensu stricto 7* was the most common genus of the interdigital area. In contrast, one previous study found that healthy canine skin (axilla, pinna, and groin) and mouth was predominantly colonized by *Porphyromonas*, *Staphylococcus*, *Streptococcus*, and *Propionibacterium*, with *Porphyromonas* significantly colonizing the axilla [14]. In another study, analysis of healthy canine skin (dorsal nose, nasal mucosa, lip commissure, conjunctiva, periocular skin, ear canal, concave pinna, dorsal lumbar area, axilla, groin, interdigital skin, and perianal area), revealed *Ralstonia* as the most abundant genus in most of the samples [13]. A recent exploratory study with six healthy dogs showed that on the skin (inguinal, axilla, periocular and trunk), *Pseudomonas* was most abundant followed by *Kocuria*, *Porphyromonas*, and *Corynebacterium* [62]. While another one identified *Propionibacterium acnes*, *Corynebacterium*, and *Porphyromonas* as most abundant in the skin (dorsal neck, axilla, and abdomen) of 40 healthy dogs [53]. These findings are in contrast to our study. *Macrococcus* is a Gram-positive coccoid bacterium, previously placed into the *Staphylococcus* genus but since 1998 assigned to its own genus [64]. It is composed of eight species that are closely related with species of the genus *Staphylococcus* [63]. Current information on the distribution of *Macrococcus* is limited. This genus is described primarily as part of the microbiota of mammals and in milk and meat according to a current review paper [63]. Even though it is considered as a non-pathogenic bacterium, there are a few reports of infections associated with *Macrococcus caseolyticus* and *M*. *canis* [64–67]. A recent study found that of 162 dogs, 13 carried *M*. *canis* and six *M*. *caseolyticus* predominately in cutaneous (axilla and groin) non-infectious sites. Six *M*. *canis* and one *M*. *caseolyticus* strains were isolated from animals with rhinitis, otitis externa, dermatitis, and mastitis [64]. As both healthy and infected skin was colonized, it was concluded that *Macrococcus* is an important opportunistic bacterium of the canine skin [64]. Because *Macrococcus* contributed mainly to the difference in bacterial community composition of the axilla between non-allergic and allergic GSDs, and was the genus with the highest relative abundance found on the skin of non-allergic dogs a potential protective role can be speculated, too. Thus, further studies regarding the distribution and role of bacteria of this genus in healthy and diseased dogs are needed.

An important finding of our study was that the site-specific bacterial composition significantly differs with allergic skin disease. The SIMPER analysis revealed the taxa with the major contribution to these changes, even though the mean relative abundance of some of those taxa (e.g., *Staphylococcus*) did not differ significantly between non-allergic and allergic GSDs. A possible explanation for this is that SIMPER analysis identified genera that had the highest contribution to differences between the groups without being necessarily significantly different. Furthermore, the axilla of allergic dogs showed a significantly decreased diversity (species richness) indicating dysbiosis (due to higher relative abundance of *Clostridium sensu stricto* 7 and *Sphingomonas*), even though the atopic dogs showed no clinical flares and had no pyoderma at the time of the sampling. This is consistent with another study of atopic dogs with pyoderma, which demonstrated different bacterial communities and reduced diversity in the allergic dogs [14]. The first 16S rRNA gene amplicon based NGS study using allergic dogs without flares, like our study population, did not show significant differences of the skin microbiota community composition in comparison with healthy dogs in contrast to our findings, but did show that allergic skin had lower diversity (richness), similar to our findings [13]. The ear canal of allergic dogs without signs of otitis also had a bacterial composition significantly different from healthy dogs’ ear canal, and without a significant difference in diversity between the groups, similar to our results [1]. A trend of dysbiosis in association with allergy was shown with significantly increased abundance of *Staphylococcus* and *Ralstonia* in the atopic ear canals [1]. In general, we were able to show a trend of dysbiosis for all the sites evaluated in total, with significantly reduction of *Macrococcus* and *Brevibacterium* and increase of the phylum Proteobacteria and the genera of *Sphingomonas* and *Nocardioides*.

Six out of 12 allergic dogs received oclacitinib, which is a Janus kinase inhibitor with anti-pruritic and anti-inflammatory properties [68]. Until now there is no study investigating the impact of oclacitinib on the skin microbiota. One study documented that treatment with cyclosporine or corticosteroids did not affect the cutaneous microbiota in dogs, evaluating these dogs before, during, and after treatment [69]. This is in line with our findings, bacterial composition of allergic dogs with and without oclacitinib did not show any significant differences. Thus, we suggest that oclacitinib may not influence the overall comparison between the non-allergic and allergic dogs, but future studies with larger population should confirm our finding.

One limitation of our study was the small sample size. However, our sample size is larger than in previous studies [1,13,62,69]. Furthermore, our study adds to a growing corpus of research that helps us better understand the microbiota inhabiting the skin and ear canal of dogs and can be used to design larger confirmatory studies. The study was a cross-sectional analysis and therefore it remains unclear if findings are a cause or a result of allergy. Regarding the description of the cutaneous microbiota only a longitudinal study could clarify if the composition of the microbiota is stable or only transient. Further studies including also non-allergic dogs from other breeds are required, in order to evaluate if the skin microbiota of non-allergic GSDs is breed specific or not. Because previous studies of humans’ skin microbiota detected a low amount of microbial DNA when standard PCR was used, a nested PCR was chosen in our study [26,27]. A bias in alpha diversity and community structure (beta diversity = has been documented due to a nested PCR in stool samples but not in vaginal swabs and might be considered as a possible limitation factor of our study [70]. However, the microbiota and DNA quantity obtained from the skin and in the ear channels of dogs by swab-based sampling is more similar to the output obtained from vaginal swabs than form stool samples which have in general a higher load of bacteria/DNA which make a nested approach for those samples unnecessary. Currently, no standard protocol exists for the methodology, and as consequence different primers are used in different studies [48]. Because there is very limited systematic comparison of the primers, and no "perfect" primer exists [71], most commonly, primers are selected based recommendations and the experimental experience of the laboratory [54]. Our second primer system targeted the V4-V5 hypervariable region, which is currently used for 16S rRNA gene sequencing-based microbial profiling [25,54,72]. Our choice was based on studies that suggest an accurate estimation of multiple taxa and an efficient phylogenetic resolution with this hypervariable region [25,54]. These advantages, were again demonstrated recently [73]. On the other hand, as it was shown in the same study, targeting this region (or targeting other regions) might have an effect on the results, because different primers do not always detect as expected specific bacterial communities, resulting in a bias when quantification of those communities is assessed [73]. But the primer system amplifying the V4-V5 was one of the primer systems with was less biased, and probably these effects may be subtle [73]. It was also shown that the analysis of bacterial community composition (beta diversity studies) were robust for the use of different primers [71,73]. In order to minimize the possibility to include a dog with a subclinical cAD into the non-allergic group, the age of the healthy dogs had to be more than four years old. Consequently, the age of dogs of two groups was significantly different. This might be a limitation, as it has been shown in humans that the skin microbiota evolves with age (first years after birth or with sexual maturation) [74–76]. In addition, all dogs were living in the same area of Germany and the environment affects skin microbiota [77], possibly leading to differences between results of studies from other countries. In our study, we did not evaluate the association of rural or urban environment with the dogs’ cutaneous microbiota. A last limitation of our study is that the 16S rRNA gene sequencing cannot differentiate dead from alive bacteria [2]. Larger studies including other breeds, several countries, as well as dogs with and without allergy flares could elucidate the canine skin microbiota in health and disease.

## Conclusion

This study describes and compares the cutaneous and ear canal microbiota of non-allergic and allergic GSDs, showing that allergic dogs have a significantly different bacterial community composition and significantly lower species richness (alpha diversity). The skin and ear canal microbiota of non-allergic GSDs is highly variable, and the ear canal has a significantly lower species richness than the body skin.

## Supporting information

S1 File (XLSX)

S2 File (DOCX)
