# Supplementary material for: Description and comparison of the skin and ear canal microbiota of non-allergic and allergic German shepherd dogs using next generation sequencing
Source: PLoS One. 2021 May 3;16(5):e0250695. doi: 10.1371/journal.pone.0250695 (PMC8092680; doi:10.1371/journal.pone.0250695)
Supplement: S2 File — (DOCX) [file pone.0250695.s002.docx]

**Supporting information S2**

Figure 1. Plots of alpha diversity indices a) Shannon (total diversity), and b) Chao 1 (number of taxa considering the number of singletons) per body site (axilla, interdigital, groin, ear canal) of healthy (non-allergic) German shepherd dogs using Chao index, based on Illumina 16S rRNA gene amplicon sequencing of microbial communities.

Figure 2. Comparative analysis of the skin microbiota composition of relative abundance patterns of six homes (two dogs per home) of 12 healthy dogs, performed by NMDS analysis based on a Bray-Curtis similarity matrix. The body site was included as environmental parameter.

Figure 3. Plots of alpha diversity indices Shannon (total diversity) and Chao1 (number of taxa considering the number of singletons) per body site (axilla, interdigital, groin, ear canal) of allergic GSD. (a) Body site comparison. No significant differences were obtained between the body sites using Kruskal-Wallis tests. (b) and (c) showing Shannon (b) and Chao1 (c) indices for individual non-allergic dog samples.

Figure 4. Comparative analysis of the bacterial community composition of the skin microbiota of all allergic dogs. Comparative analysis of the relative abundance patterns of the skin microbiota composition of all body sites (different objects), performed by NMDS analysis based on a Bray-Curtis similarity matrix. The sex, body site, and home of living were included as environmental parameters (biplots).

Figure 5. NMDS plots of allergic dogs grouped into subgroups according to antipruritic treatment, allergic with oclacitinib (n=6) and without oclacitinib (n=6), respectively.

Figure 6. Comparative analysis of the skin microbiota composition of the ear canal (O) between healthy and allergic German shepherd dogs performed by NMDS analysis based on a Bray-Curtis similarity matrix at the genus level. The health status was included as environmental parameter (A) and body site (B).

Figure 7 Forward step redundancy analysis (RDA) indicating the impact of metadata on alpha diversity (Shannon index a, b; Chao1, c, d) including all studied dogs and body skin sites (a,c all factors included; b/d, excluding the factor household).
